# Evaluation of the executive dysfunction in patients with bipolar disorder

**DOI:** 10.1192/j.eurpsy.2023.1481

**Published:** 2023-07-19

**Authors:** O. Vasiliu

**Affiliations:** Psychiatry Department, Dr. Carol Davila University Emergency Central Military Hospital, Bucharest, Romania

## Abstract

**Introduction:**

Bipolar disorders (BD) associate impairments in cognitive functioning not restricted to acute mood episodes. The functional impact of these cognitive dysfunctions is significant in many cases, therefore, an extensive evaluation is granted for BD patients from this perspective. Executive dysfunction in BD has been reported, especially during mania and was associated with formal thought disorder, but multiple cognitive deficits may reflect an underlying pathologic process related to the mood disorder itself.

**Objectives:**

To assess the level of executive dysfunction in a sample of patients with type I BD (BDI) using structured measurement methods.

**Methods:**

Five patients diagnosed with BDI (two patients during manic episodes, one during the euthymic phase, and 2 during depressive episodes) were evaluated for working memory (n-back test), verbal fluency (Controlled Oral Word Association Test, COWAT) and verbal learning (Verbal Learning and Memory test, VLMT). All patients were adults, mean age of 35.5 years, and all were undergoing psychotropic treatment with antipsychotics and/or mood stabilizers. Also, five normal healthy controls, matched for age, were evaluated using the same test battery.

**Results:**

All five BDI patients presented dysfunctions (three at a trend level, and one at a significant level, p<0. 01) in at least one cognitive domain, with three patients presenting modifications in all three tests that were administered vs. normal controls. The most affected domain was verbal learning, followed by working memory and verbal fluency. No individual in the control group had significant cognitive deterioration, on any outcome assessed.

**Conclusions:**

Cognitive domains are altered in BDI patients, therefore, a test battery destined to quantify executive dysfunctions, independent of the presence of an acute mood episode or during the euthymic phase, may be useful for case management.

**Disclosure of Interest:**

None Declared

